# Frequent Amplification of CENPF, GMNN and CDK13 Genes in Hepatocellular Carcinomas

**DOI:** 10.1371/journal.pone.0043223

**Published:** 2012-08-13

**Authors:** Hye-Eun Kim, Dae-Ghon Kim, Kyung Jin Lee, Jang Geun Son, Min-Young Song, Young-Mi Park, Jae-Jung Kim, Sung-Won Cho, Sung-Gil Chi, Hyun Sub Cheong, Hyoung Doo Shin, Sang-Wook Lee, Jong-Keuk Lee

**Affiliations:** 1 Asan Institute for Life Sciences, University of Ulsan College of Medicine, Seoul, Korea; 2 Department of Internal Medicine, Chonbuk National University Medical School and Hospital, Chonju, Chonbuk, Korea; 3 Ajou University School of Medicine, Suwon, Korea; 4 School of Life Sciences and Biotechnology, Korea University, Seoul, Korea; 5 SNP-genetics, Seoul, Korea; 6 Department of Life Science, Sogang University, Seoul, Korea; 7 Department of Radiation Oncology, University of Ulsan College of Medicine, Seoul, Korea; National Cancer Center Research Institute, Japan

## Abstract

Genomic changes frequently occur in cancer cells during tumorigenesis from normal cells. Using the Illumina Human NS-12 single-nucleotide polymorphism (SNP) chip to screen for gene copy number changes in primary hepatocellular carcinomas (HCCs), we initially detected amplification of 35 genes from four genomic regions (1q21–41, 6p21.2–24.1, 7p13 and 8q13–23). By integrated screening of these genes for both DNA copy number and gene expression in HCC and colorectal cancer, we selected *CENPF* (centromere protein F/mitosin), *GMNN* (geminin, DNA replication inhibitor), *CDK13* (cyclin-dependent kinase 13), and *FAM82B* (family with sequence similarity 82, member B) as common cancer genes. Each gene exhibited an amplification frequency of ∼30% (range, 20–50%) in primary HCC (n = 57) and colorectal cancer (n = 12), as well as in a panel of human cancer cell lines (n = 70). Clonogenic and invasion assays of NIH3T3 cells transfected with each of the four amplified genes showed that *CENPF*, *GMNN*, and *CDK13* were highly oncogenic whereas *FAM82B* was not. Interestingly, the oncogenic activity of these genes (excluding *FAM82B*) was highly correlated with gene-copy numbers in tumor samples (correlation coefficient, r>0.423), indicating that amplifications of *CENPF*, *GMNN*, and *CDK13* genes are tightly linked and coincident in tumors. Furthermore, we confirmed that *CDK13* gene copy number was significantly associated with clinical onset age in patients with HCC (*P = *0.0037). Taken together, our results suggest that coincidently amplified *CDK13*, *GMNN*, and *CENPF* genes can play a role as common cancer-driver genes in human cancers.

## Introduction

Tumors are characterized by a complex pattern of cytogenetic and genetic alterations, including somatic mutations, chromosomal rearrangements, and copy-number changes [1], [2]. Genetic changes in the cancer cell genome lead to aberrant regulation of cell proliferation, apoptosis, genome stability, angiogenesis, invasion, and metastasis during the genesis and progression of tumors. An increase in gene copy number in the cancer cell genome, called gene amplification, is a particularly common mechanism by which the expression levels of genes that contribute to cancer development are regulated [3]. In addition, DNA copy number alterations may provide important clues in identifying tumor suppressor genes and oncogenes [4]. According to a recent update, at least 77 amplified and overexpressed genes are involved in the development of human cancer as oncogenes [5]. Although genomically amplified regions are very common in human cancer genomes, it is often difficult to identify the true cancer gene on such amplicons, which often contain multiple genes. Therefore, the identification of true oncogenes in regions amplified in cancer is essential for a better understanding of the pathogenesis of cancer and for the development of clinical applications [6].

Hepatocellular carcinoma (HCC) is one of the most common types of cancer and is a prevalent human malignancy worldwide. Approximately 80% of all HCC cases have been linked to three main causative agents: hepatitis B virus (HBV), hepatitis C virus (HCV), and aflatoxin B1 [7], [8]. Prolonged exposure to these agents is thought to cause accumulated chromosomal aberrations and altered gene expression, eventually resulting in HCC tumor progression [7]. Although frequent genomic aberrations have been described in many human HCCs [8], [9], the specific genomic alterations and genes that drive HCC development are not fully understood.

Previous studies have demonstrated that single-nucleotide polymorphism (SNP) genotyping technology can be used to detect copy-number variations [10]–[12]. In this study, we performed a SNP-based genome-wide screening of gene amplification using the Illumina Human NS-12 BeadChip to identify amplified and overexpressed genes in primary HCC. Our results suggest that integrating DNA copy number and gene expression data from tumors will help to identify potential cancer-driver genes that exert their oncogenic functions through a gene-amplification mechanism.

## Results

### SNP-based genome-wide screening of amplified genes with high mRNA expression in HCC

**Figure 1:**
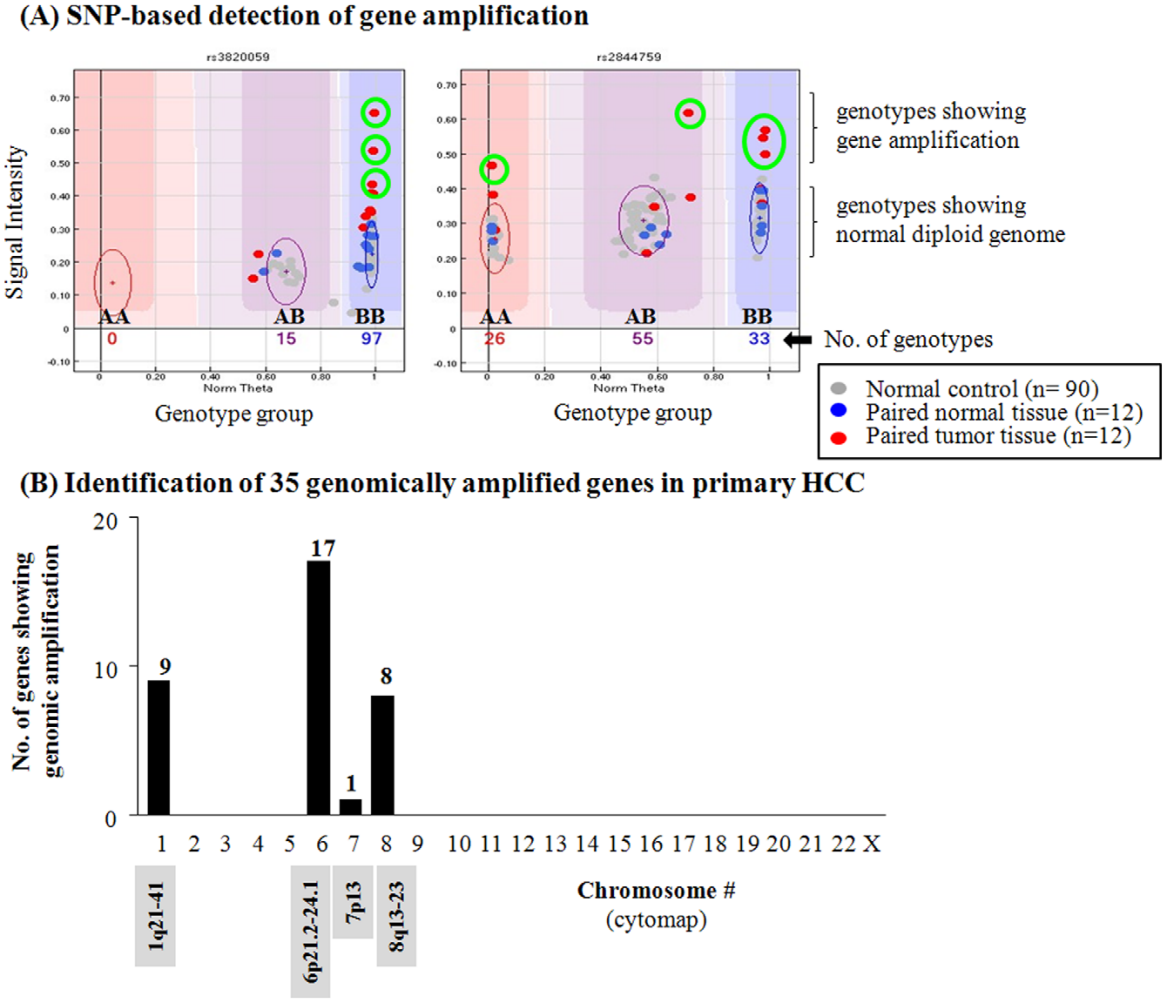
SNP-based detection of gene amplification in 12 paired primary HCC tissues using a Human NS-12K SNP chip. Gene amplification was detected based on genotype intensities that were more than 1.5-fold higher in tumor samples (shown in red dots) than in normal samples (shown in blue dots) in each genotype group (X-axis), with manual confirmation of intensity and the images of genotype cluster plots (A). A total of 90 normal genome samples isolated from the blood of healthy individuals were used as controls (shown in gray dots) for comparison. The samples showing gene amplification were marked with green circle in the genotype cluster images. The numbers shown inside genotype cluster plots indicate the numbers of genotype for specific SNP. Y-axis and X-axis indicate the signal intensity and genotype group, respectively. Using the Human NS-12K SNP chip, a total of 35 genomically amplified genes were initially identified in primary HCC samples (B). A detailed gene list is presented in Table S1.

**Figure 2:**
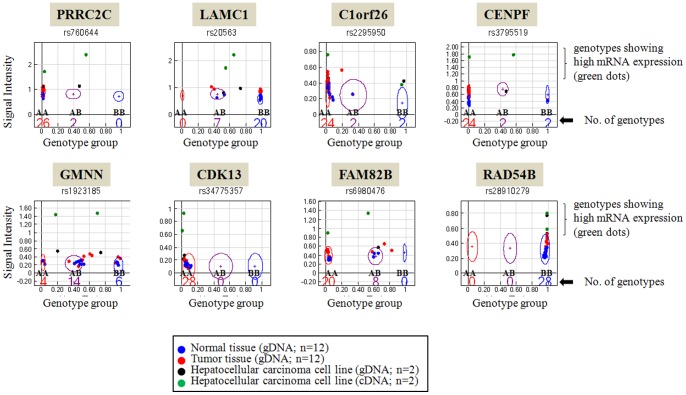
Selection of eight amplified genes with high mRNA expression from among 35 genomically amplified genes in primary HCC tissues. Human NS-12K SNP chip genotyping experiments was performed using cDNA templates derived from two hepatoma cell lines. Each dot indicates a single DNA sample; samples tested: 12 normal tissue gDNAs (blue), 12 tumor tissue gDNAs (red), two HCC cell line gDNAs (black), and two HCC cell line cDNAs (green). The numbers shown inside genotype cluster plots indicate the numbers of genotype for specific SNP. In addition, high mRNA expression (shown in green dots) was detected with high signal intensity in the genotype cluster plots. Y-axis and X-axis indicate the signal intensity and genotype group, respectively.

To identify genetic abnormalities in primary HCC, we performed SNP genotyping using genomic DNA isolated from primary HCC tissues (n = 12 paired tumor samples). By examining the SNP genotype intensity and genotype cluster plots, we were able to identify 35 amplified genes from four genomic regions (1q21–41, 6p21.2–24.1, 7p13 and 8q13–23) in primary HCC samples (Figure 1; Table S1). Each locus contained multiple amplified genes except 7p13, indicating that gene amplification in these regions is mainly attributable to an increase in chromosomal copy numbers. To determine the mRNA gene expression of the genomically amplified genes in liver cancer, we initially examined liver cancer cell lines using Human NS-12K BeadChip genotyping assays with cDNA templates derived from two HCC cell lines (Figure 2). Among the 35 genomically amplified genes identified in primary HCC, we found that 8 genes were genomically amplified and functionally overexpressed in HCC. The eight genes were *PRRC2C* (proline-rich coiled-coil 2C), *LAMC1* (laminin, gamma 1), *C1orf26* (chromosome 1 open reading frame 26), *CENPF* (centromere protein F, 350/400 kDa/mitosin), *GMNN* (geminin, DNA replication inhibitor), *CDK13* (cyclin-dependent kinase 13), *FAM82B* (family with sequence similarity 82, member B), and *RAD54B* (RAD54 homolog B [*S. cerevisiae*]). Interestingly, these eight genes were mostly associated with tumorigenic effects, particularly in relation to cell-cycle regulation or DNA repair.

### Identification of CENPF, GMNN, CDK13, and FAM82B as frequently amplified genes common to both HCC and colorectal cancer

**Figure 3:**
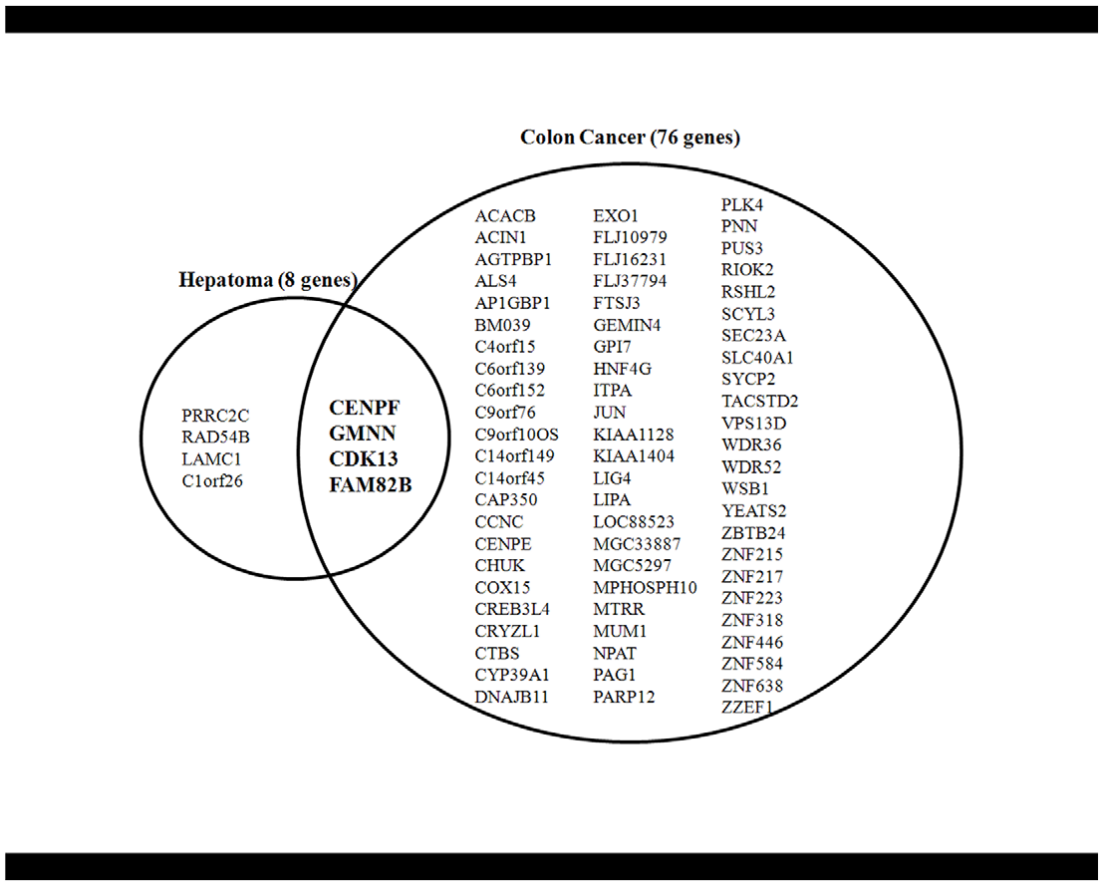
Detection of amplifed genes common to both liver and colon cancer. Identification of overlapping amplified and overexpressed genes in liver (n = 8 genes) and colon cancer (n = 76 genes) pinpointed *CENPF*, *GMNN*, *CDK13*, and *FAM82* as candidate cancer genes.

**Figure 4:**
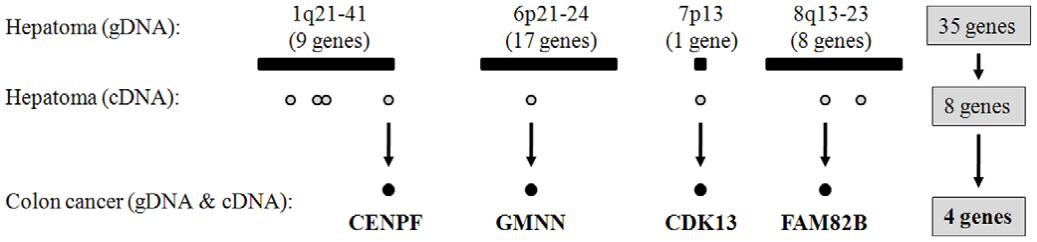
Detection of four amplified genes with high mRNA expression common to both liver and colon cancer. Among 35 amplified genes in HCC, 8 genes were initially selected as HCC-specifically amplified and overexpressed genes and then four genes (*CENPF*, *GMNN*, *CDK13*, and *FAM82B*) were ultimately selected as amplified and overexpressed cancer genes common to both liver and colon cancer.

**Figure 5:**
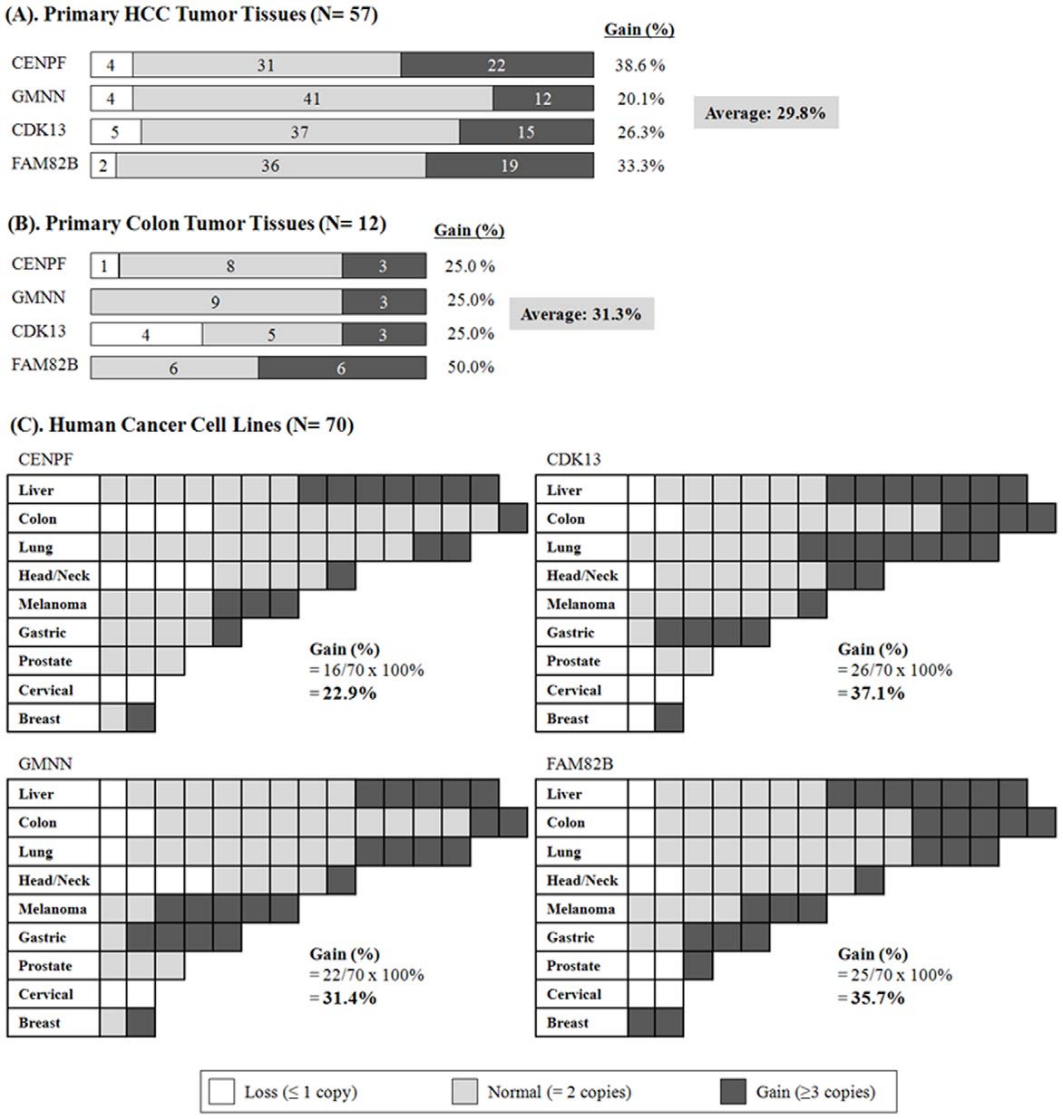
Screening of gene amplification in primary tumor tissues and a panel of human cancer cell lines. The number of gene copies in primary liver tumor tissues (n = 57) and colon tumor tissues (n = 12) was examined using the TaqMan Copy Number Assay System, as described in Materials and Methods. All paired nontumor tissues had a normal number of gene copies (2 copies) in both HCC and colorectal cancer patients. However, a various numbers of gene copies, especially gene amplification, were detected in the primary liver tumor tissues (A) and colon tumor tissues (B). In addition, copy numbers were also examined in a panel of human cancer cell lines (n = 70) (C).

**Figure 6:**
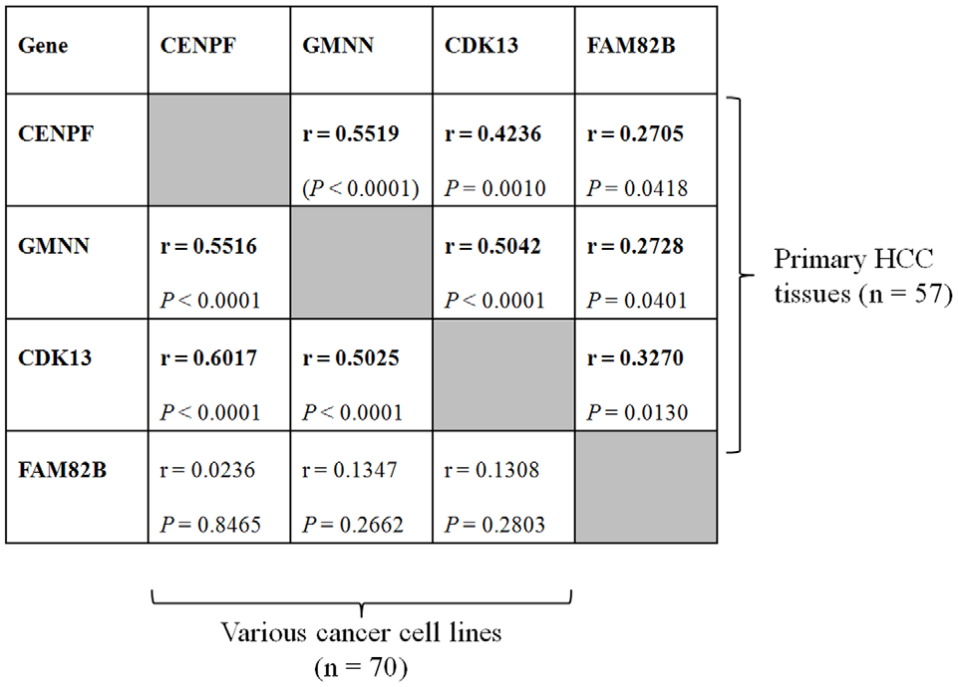
Correlation of gene copy number between four amplified genes in primary HCC tissues and various cancer cell lines. Pearson Correlation coefficients and *P*-values were determined as described in the Materials and Methods. Above the diagonal indicates primary HCC tissues (n = 57 primary tumor tissues) and below the diagonal indicates various cancer cell lines (n = 70 cancer cell lines from nine different cancer types).

To identify the true cancer genes within the amplified regions in primary HCC, we examined the increase in gene copy numbers in another type of tumor, namely colon cancer, using the same SNP chip genotyping platform. Using colon cancer cell lines derived from colon cancer patients (n = 11), we identified a total of 76 genomically amplified genes that were highly expressed at the mRNA level (Figure 3). Of these, the following four genes were common to both HCC and colon cancer: *CENPF*, *GMNN*, *CDK13*, and *FAM82B* (Figure 4). The amplification of those four genes was also confirmed in primary HCC (n = 57 tumor tissues) and colon cancer (n = 12 colon tumor tissues) using the TaqMan Copy Number Assay system. On average, each gene was amplified in approximately 30% of primary liver and colon tumor tissues (Figure 5A & B). To further confirm this finding, we screened a panel of 70 human cancer cell lines from nine different types of cancer cell lines for amplification of the selected four genes using the TaqMan Copy Number Assay (Figure 5C). On average, the *CENPF* gene was amplified in 22.9% of cancer cell lines, and showed a particularly high amplification frequency in liver cancer cell lines (50%; n = 7/14). The *GMNN* gene was amplified in 31.4% of cancer cell lines, with very high amplification rates in melanoma (71%; n = 5/7) and gastric cancer cell lines (80%; n = 4/5). *CDK13* and *FAM82B* genes were amplified in 37.1% and 35.7% of cancer cell lines, respectively (Figure 5C). Collectively, these data indicate that amplification of these four genes frequently occurs not only in HCC and colorectal cancer but also in many other types of human cancers. Moreover, differential gene expression data in the ONCOMINE database (www.oncomine.org) showed that these four amplified genes exhibited differentially high gene expression in cancer tissues (Figure S1 and Figure S2), confirming that these genes can be genomically amplified and overexpressed in many cancer types. However, no somatic mutations were detected in primary HCC tumor samples (data not shown), suggesting that somatic point mutations do not underlie the tumorigenic effects of the four amplified genes. Interestingly, with the exception of the *FAM82B* gene, amplification of these genes was tightly linked and coincident in various cancers (correlation coefficient, *r*>0.423 for gene-copy numbers between genes; Figure 6).

### Oncogenic and clinicopathologic effects of CENPF, GMNN and CDK13, but not FAM82B, in HCC

**Figure 7:**
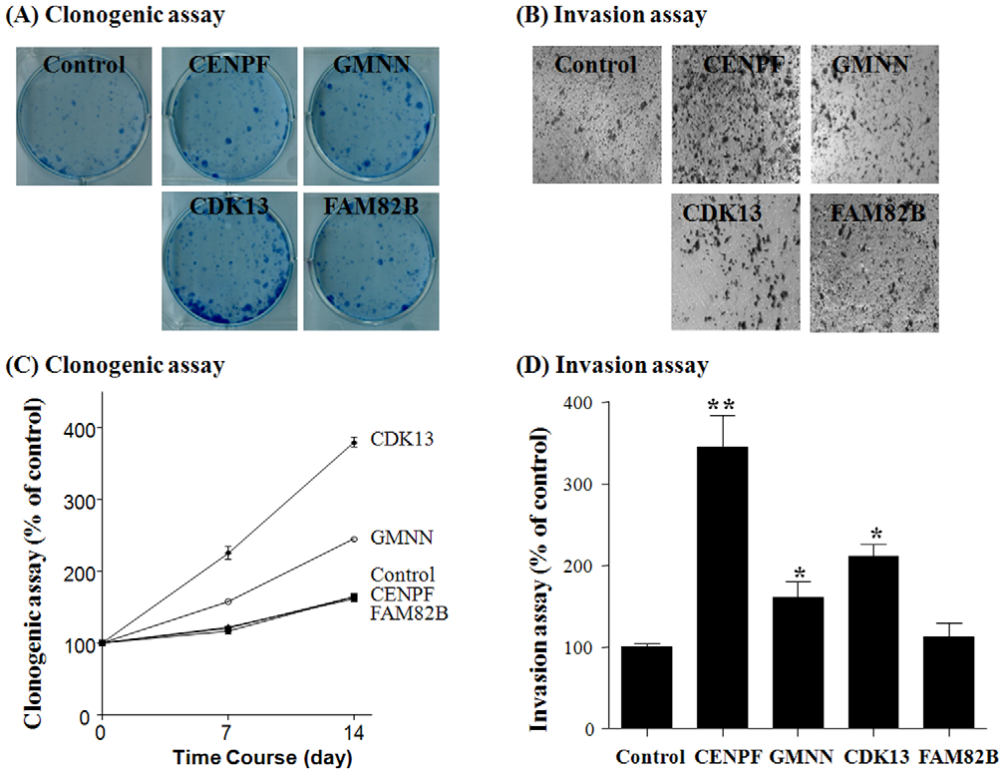
Oncogenic effects of *CENPF*, *GMNN* and *CDK13* after stable transfection in NIH3T3 cells. Clonogenic assays and invasion assays were performed using stably transfected NIH3T3 cells with *CENPF*, *GMNN*, *CDK13* or *FAM82B* gene. The images of clonogenic assays (A) and migration assays (B) in a representative experiment were taken at day 14 and 24 h after treatment, respectively, as described in Materials and Methods. Data representing means and standard deviations of triplicate experiments are shown for (C) clonogenic assays and (D) invasion assays as described in Materials and Methods (*P<0.05, **P<0.01 versus control group; t-test).

**Figure 8:**
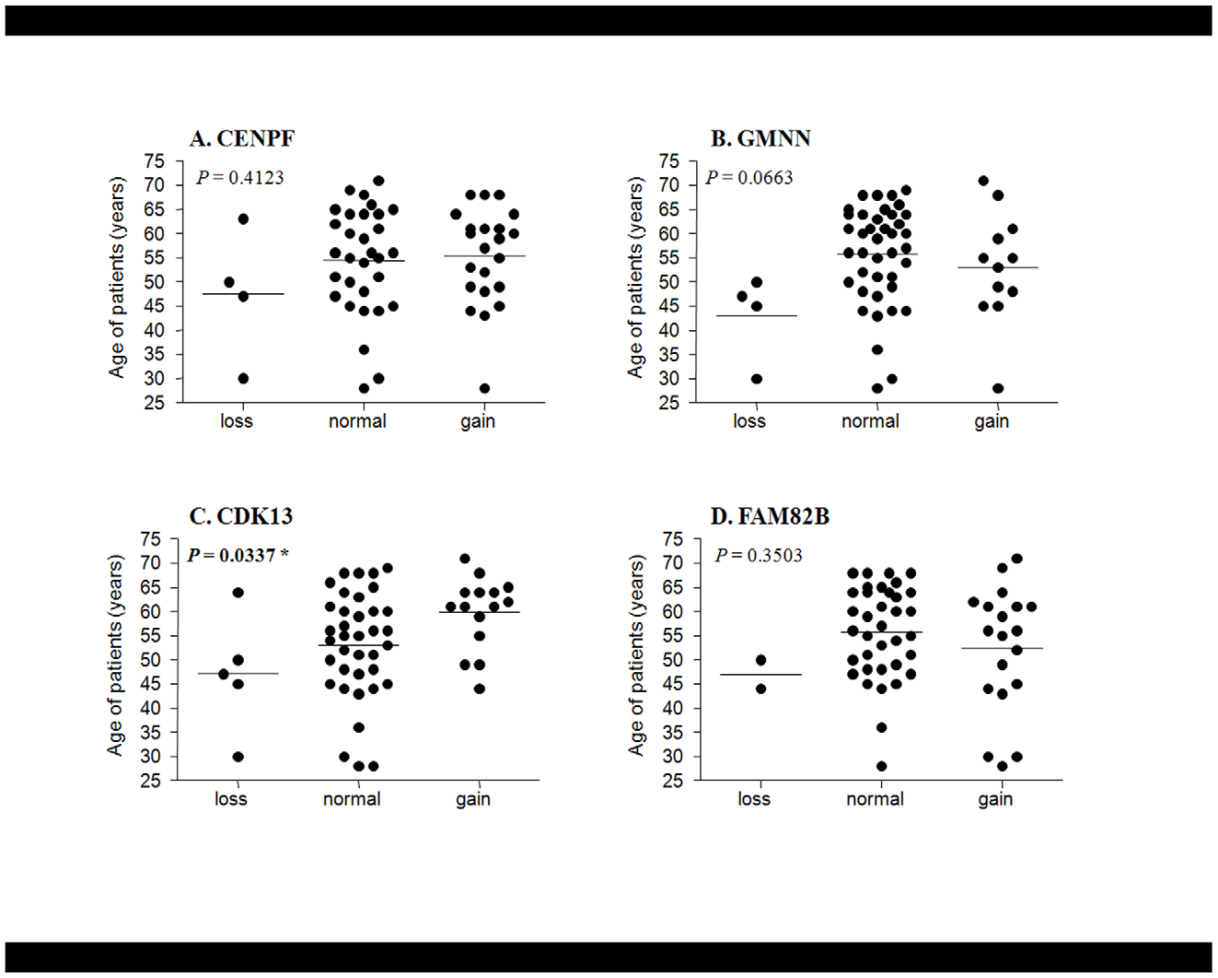
Association of *CDK13* gene amplification with clinical onset of cancer in HCC patients. Mean age of clinical onset is indicated by horizontal bars. The statistical significance of differences was determined by ANOVA.

To investigate the tumorigenic effects of the encoded products of these four amplified genes, we performed clonogenic and invasion assays in NIH3T3 cells stably transfected with expression plasmids for the corresponding gene. Interestingly, we observed a significant increase in colony formation activity, *in vitro* cell proliferation activity and/or migration ability in NIH3T3 cells stably expressing *CDK13*, *GMNN* or *CENPF* genes, but not in those expressing the *FAM82B* gene (Figure 7 and Figure S3). Specifically, *CDK13* expression promoted strong colony forming activity and an intermediate degree of migration activity, whereas *GMNN* expression led to an intermediate amount of colony-forming activity. Although *CENPF*-expressing cells did not exhibit colony-forming activity, they did show very strong migration activity. In contrast, the *FAM82B* gene was not significantly associated with oncogenic activity. Additionally, to determine the effects of gene amplification on anticancer drug sensitivity, we performed *in vitro* cell viability assays using a panel of HCC cell lines containing various copy numbers of the four amplified genes. HCC cell lines in which *CENPF* and *CDK13* were amplified were more resistant to 5-FU (*P* = 0.044) and doxorubicin (*P* = 0.046) treatment (Table S2). However, HCC cell lines with *CDK13* amplifications were slightly susceptible to tamoxifen treatment (*P* = 0.045). These data suggest that amplification of *CDK13* and *CENPF* genes also plays a role in the therapeutic response to anticancer drug treatment. Finally, we analyzed the association of gene amplification with clinical characteristics of HCC patients, including age, AFP level, tumor grade, and tumor size. Interestingly, we found that CDK13 gene amplification status was associated with the age of clinical onset of HCC (*P* = 0.0337; Figure 8). GMNN gene amplification also showed marginal significance with the age of clinical onset of HCC (*P* = 0.0663; Figure 8) and tumor size (*P* = 0.0899; data not shown). However, other gene amplification status was not associated with any clininopathological parameters (data not shown). Collectively, these results suggest that *CENPF*, *GMNN*, and *CDK13* play a role in the tumorigenesis of human cancers.

## Discussion

Carcinogenesis results in the accumulation of multiple genetic alterations, including loss of heterozygosity (LOH), somatic mutations, and/or gene amplification [2]. These changes can affect a variety of genes, several of which are involved in deregulation of intracellular signal transduction and the cell cycle, and ultimately lead to uncontrolled proliferation of cancerous cells. Gene amplification, in particular, is a key genetic event in cancer [5]. In this study, using Illumina Human NS-12 BeadChips containing approximately 12,000 nonsynonymous coding SNPs to perform an integrated genome-wide screening of DNA copy number and gene expression, we pinpointed four genes – *CENPF*, *GMNN*, *CDK13* and *FAM82B* – that were amplified and overexpressed in both HCC and colorectal cancer. These four genes were found to be amplified and overexpressed not only in HCC and colorectal cancer, but also in many other types of human cancer, as confirmed by screening a panel of human cancer cell lines and searching the ONCOMINE database (Figure S1 and Figure S2). As shown in the Figure S2, we found that 5.0 fold and 3.4 fold increases of *CENPF* and *GMNN* gene expression were observed in liver cancer tissues, respectively. We also found that exogenous, stable expression of *CENPF*, *GMNN* and *CDK13*, but not *FAM82B*, in NIH3T3 cells exerted strong tumorigenic effects (Figure 7). Notably, the gene-amplification status of *CDK13*, *GMNN*, and *CENPF* genes was also significantly associated with clinical pathophysiological effects in HCC. Taken together, our results indicate that the integrated screening of gene copy number and gene expression in multiple tumor types is an efficient strategy for the identification of potential common cancer genes.

It has been previously reported that *CENPF* is highly expressed in breast tumors of patients with poor prognosis and is associated with proliferation in different human malignancies [13]. In addition, *GMNN* has been reported to be an inhibitor of cell proliferation and a potential tumor suppressor gene that is expressed at high levels in various cancer cell lines and in primary tumors [14]. In addition, the overexpression of geminin stimulated cell cycle progression and proliferation in both normal cells and cancer cells, leading to aneuploidy [14], [15]. Although *CDK13* and *FAM82B* have not been well characterized in this context, they are associated with cell proliferation and chromosome stability in various cancer types [16], [17]. Thus, the four amplified genes identified here are involved in cell-cycle regulation and/or cell proliferation, suggesting that amplification of genes involved in these cellular processes is a key mechanism for tumorigenesis. The high frequency with which these genes are amplified in both HCC (∼30% of HCC tumors) and colorectal cancer (∼30% of colorectal tumors) as well as functional evidence for their clinicopathologic effects and/or transforming activities in stably transfected NIH3T3 cells, suggests that the amplified genes identified here work as drivers of tumorigenesis. The exception to this is *FAM82B*, which showed no clonogenicity or invasion activity upon expression in NIH3T3 cells and thus does not appear to play an important role as a cancer-driver gene. Furthermore, the high correlation between copy numbers of *CENPF*, *GMNN*, and *CDK13* suggests that these three amplified genes utilize and/or share the same tumor-development pathway. This interpretation is supported by a previous report showing that geminin is critical for continued DNA synthesis and entering cells to cell-cycle progression from G2 to M phase via cyclin-dependent kinase (CDK) genes [18].

The cyclin-dependent kinase *CDK13* gene, in particular, is a novel oncogene with potent oncogenic activity, as confirmed in our study. Interestingly, a loss of *CDK13* gene copies was significantly associated with early-onset of HCC, whereas high gene-copy numbers (i.e., amplification) was significantly associated with late-onset of HCC. These data suggest that the initial loss of *CDK13* may trigger amplification of the *CDK13* gene, since loss of DNA replication control is a potent inducer of gene amplification [19]. In addition, because no somatic point mutations in *CDK13*, *GMNN,* or *CENPF* genes were detected in primary tumor tissues, we think that the oncogenic effects of these genes are mainly due to alterations in gene copy numbers, particularly gene amplification. Our results suggest that inhibition of the amplified genes, *CENPF*, *GMNN* and *CDK13*, might be an effective new therapeutic strategy against human cancer. Consistent with this, a previous study has demonstrated that suppression of geminin activity selectively kills cancer cells [20]. Furthermore, inhibitors of *CDK13* could be promising anticancer therapeutics since targeting kinases in the cancer genome is a major strategy in anticancer-drug development.

In this study, we mainly focused on genes that were amplified and overexpressed in common among multiple cancer types. However, other gene(s) in the same amplicons could also play a role in tumor development in specific types of cancer through synergistic or independent actions. For example, amplification and overexpression of the *PRRC2C* gene, located in the same amplicon as the *CENPF* gene, have been observed in primary HCC. It has also been reported that the *PRRC2C* gene is amplified and overexpressed in approximately 30% of bladder cancers [21], although our subsequent validation experiments eliminated this gene as a common amplified and overexpressed cancer gene. On the other hand, liver cancer is one of the leading causes of cancer-related deaths mainly because of the high incidence of HBV infection in Asia, including Korea. Therefore, we cannot rule out a possible role of HBV in gene amplification in HCC, since HBV-mediated mutagenesis has been frequently observed in human liver cancers [22].

In summary, we demonstrated that a high-density SNP chip, particularly one oriented toward encoded genes, is a very powerful tool for detecting amplified and overexpressed genes in primary tumor tissues. We also found that three genes – *CENPF*, *GMNN* and *CDK13* – were frequently amplified in various human cancers and were significantly associated with several pathophysiological phenotypes in HCC. Notably, *CDK13* amplification was significantly associated with the clinical onset of HCC and also showed a strong tumorigenic effect *in vitro*. The identification of the amplified genes *CENPF*, *GMNN*, and *CDK13* in tumors provides new insight into the carcinogenesis process, and suggests that these genes can be used as novel cancer markers for the development of diagnostic, prognostic, and/or targeted therapies.

## Materials and Methods

### Ethics Statement

The study protocol conformed to the ethical guidelines of the Institutional Review Board with the approval number 2008–0042, and written informed consent was obtained from each patient.

### Tumor cell lines and tumor tissue samples from cancer patients

A total of 12 HCC cell lines and 11 colorectal cancer cell lines originating from Korean cancer patients [23]–[25] were obtained from the Korean Cell Line Bank (http://cellbank.snu.ac.kr). Cancer cell lines were maintained in RPMI-1640 medium (plus L-glutamine, 25 mM HEPES) supplemented with 10% fetal bovine serum (FBS) and antibiotics (100 U/ml penicillin, 100 µg/ml streptomycin). Samples of primary tumor tissue and surrounding nontumor liver tissues were obtained from 57 HCC patients by surgical resection in operations performed at Chonbuk National University Hospital (Chonju, Korea). A total of 12 primary colorectal cancer tissues and surrounding nontumor tissues were also obtained from Asan Medical Center (Seoul, Korea). Diagnoses were confirmed by histological examination of primary HCC and colorectal tissues by pathologists at Chonbuk National University Hospital and Asan Medical Center, respectively.

### DNA and RNA extraction

Genomic DNA and total RNA were isolated for genomic and gene analyses. Genomic DNA was extracted from primary cancer tissues and cultured cancer cells using QIAamp DNA Mini and Blood Mini kits (Qiagen, Germany). Total RNA was isolated from primary tissues and various cancer cell lines with TRIZOL reagent according to the protocols provided by the manufacturer (Invitrogen, Carlsbad, CA, USA).

### SNP genotyping

SNP genotypes of 12 primary tumor tissues as well as matching nontumor tissues were determined using the Human NS-12 BeadChip (Illumina, San Diego, CA, USA). Analyses were performed using 200 ng genomic DNA or cDNA templates derived from 10 µg of total RNA according to the manufacturer's instructions. The NS-12 BeadChip contains over 11,000 SNPs in annotated exons and untranslated mRNA regions of 6310 genes, corresponding to all known SNPs with a minor allele frequency >1% at the time the original BeadChip was designed [26]. In addition to the genome-wide coverage of SNPs in coding regions of genes, the BeadChips contain about 2,000 SNPs in introns and flanking regions of genes. Genotype clustering and calling were performed using BeadStudio software (Illumina). Gene amplifications were identified based on SNP genotypes with an intensity in tumor samples more than 1.5-fold greater than that in normal samples (90 normal control genomes and 12 normal tissues isolated from cancer patients) and were subsequently checked manually for intensity and images of genotype cluster plots.

### Determination of CENPF, GMNN, CDK13 and FAM82B copy number by quantitative real-time PCR

The number of copies of *CENPF* (centromere protein F/mitosin), *GMNN* (geminin, DNA replication inhibitor), *CDK13* (cyclin-dependent kinase 13), and *FAM82B* (family with sequence similarity 82, member B) in tumor cell lines and tumor tissue samples from cancer patients was determined by quantitative real-time polymerase chain reaction (PCR) using TaqMan Copy Number Assays (Hs03027722_cn, Hs02853781_cn, Hs01043269_cn and Hs01088078_cn; Applied Biosystems, Foster City, CA, US). The Copy Number Assay, containing two primers and a FAM dye-labeled MGB probe, detects the target gene or genomic sequence of interest, and the Reference Assay (RNase P, PN 4403326), containing two primers and a VIC dye-labeled TAMRA probe, detects a sequence that is known to exist in two copies in a diploid genome. TaqMan Copy Number Assays were performed on the 7900 HT Sequence Detection System (Applied Biosystems, Foster City, CA, USA) with sequence detection software version 2.4. Amplification mixtures (10 µl) for each target gene contained template DNA (10 ng/µl), 2X TaqMan Universal Master Mix (No AmpErase UNG), 20X TaqMan Copy Number Assay reagent, and 20X TaqMan Copy Number Reference Assay reagent in a 384-well plate. The cycling conditions were 10 min at 95°C, followed by 40 cycles of 15 s at 95°C and 60 s at 60°C. After running each experiment in quadruplicate, data files containing the sample replicate C_T_ values for each reporter dye were exported from the real-time PCR instrument software into CopyCaller Software, which is an analysis tool for PCR data analysis that calculates sample copy number values based on relative quantitation.

### Mutation screening of CENPF, GMNN, CDK13, and FAM82B

Primary tumor tissues isolated from cancer patients were screened for somatic mutations in the amplified genes (*CENPF*, *GMNN*, *CDK13*, and *FAM82B*). Primers were designed to cover all coding sequences and splice junctions of the selected genes (all primer sequences and reaction conditions are available on request). Mutation screening employed PCR followed by direct sequencing using BigDye terminator chemistry (Applied Biosystems) and analysis on an ABI 3730 DNA sequencer (Applied Biosystems). DNA polymorphisms were identified using the PolyPhred program (http://droog.gs.washington.edu/PolyPhred.html) after sequence chromatograms were base-called with the Phred program and assembled with Phrap [27].

### Real time PCR for mRNA expression

Total RNA was extracted from cells using the RNeasy kit (Qiagen, Valencia, CA) and NucleoSpin® (MACHEREY-NAGEL, Düren, Germany). RNAs were reverse-transcribed using IScript^TM^ cDNA Synthesis kit (Bio-Rad Laboratories, Hercules, CA). Real-time PCR was performed in an iCycler iQ system (Bio-Rad Laboratories) using the iQ^TM^ SYBR® Green Supermix (Bio-Rad Laboratories). The expression levels of Bax and Bcl-2 in the exposed cells were compared to those in control cells at each time point using the comparative cycle threshold (Ct) method. Fold induction was calculated by 2(−ΔΔC(T)) method [28], using the expression of ribosomal protein S18 as the reference. Data are presented as the means of triplicate samples ± SD. Primers used were listed in Table S3.

### Construction of expression vectors and stable transfection

Expression vectors for *CDK13*, *GMNN*, *FAM82B*, and *CENPF* were constructed by amplifying the coding region of each gene from human cerebral cortical cDNA (Clontech, USA) by PCR using a forward primer containing an *Eco*RI restriction site and a reverse primer with a *Not*I restriction site. The resulting single PCR products were inserted into the pcDNA4 plasmid (Invitrogen, Carlsbad, CA, USA). The integrity of the insert was confirmed by sequencing. NIH3T3 cells were transfected with recombinant expression vector or empty vector (control) using the Polyfect reagent (Qiagen, Valencia, CA, USA) according to methods recommended by the manufacturer. Forty-eight hours after transfection, cells were incubated with complete medium containing 200 μg/ml of G418 for 5 wk. High expression of transfected genes was confirmed by RT-PCR analysis. Clones resistant to G418 that showed high expression of the transfected gene were isolated and used for clonogenic and invasion assays.

### Clonogenic assay

Each stable cell line (1×10^3^ cells) was plated on 60-mm dishes containing 1% agar in 1X medium. On the following day, cells were washed twice with phosphate-buffered saline (PBS) and incubated in a 5% CO_2_ incubator at 37°C for 7–14 days. The resulting colonies were fixed with methanol and stained with crystal violet (0.1% in methanol). After aspiration of supernatant, the dye was dissolved by adding an acidic isopropanol solution (1 ml/well) and the optical density in each plate was subsequently measured at 570 nm with an ELISA (enzyme-linked immunosorbent assay) reader. Each experiment was performed in triplicate.

### Invasion assay

*In vitro* invasion assays were performed using a 24-well Transwell unit (8 μm pore size) fitted with polyvinylpyrrolidone-free polycarbonate (PVPF) filters coated with 500 μg/ml of Matrigel, as previously described [29]. The coated filters were washed thoroughly in PBS and dried immediately before use. Cells of each stable cell line were placed in the upper part of the transwell plate and incubated for 24 h at 37°C. The cells that invaded to the lower surface of the membrane were fixed with methanol and stained with 0.5% crystal violet for 10 min. The supernatant was aspirated and 0.3 ml/well of an acidic isopropanol solution was added to each well to dissolve the dye, after which the optical densities of the solutions in each well were measured at 570 nm with an ELISA reader. Cells that had migrated to the lower side of the filter were also imaged using a microscope at 200× magnification.

### Cell viability assay for stable transfectants and anticancer drug sensitivity

For cell proliferation assay using stably transfected cells, cells were seeded at a density of 1×10^4^ cells/500 μL in 48-well plates and cell viability was determined by the conventional MTT (3-[4,5-dimethythiazol-2-yl]-2,5-diphenyl tetrazolium bromide) reduction assay for 72 hr. MTT is tetrazolium salt cleaved to formazan by the mitochondrial respiratory chain enzyme succinate dehydrogenase, which is active in live cells. After incubation, cells were incubated with the MTT solution (0.2 μg/mL) for 1 h. The dark blue formazan crystals formed in intact cells were solubilized with dimethyl sulfoxide (DMSO) and absorbance was measured at 570 nm using a microplate reader (Varioskan, Thermo Electron). Cell viability (%) was calculated based on the relative absorbance compare to that of the controls. For cell viability assay for anticancer drug sensitivity, human HCC cell lines (SNU-182, SNU-354, SNU-368, SNU-398, SNU-423, SNU-449, SNU-475, SNU-739, SNU-761, SNU-878, and SNU-886) were cultured in RPMI 1640 medium (plus L-glutamine, 25 mM HEPES) supplemented with 10% FBS and antibiotics (100 U/ml penicillin, 100 µg/ml streptomycin) at 37°C in a humidified 5% CO_2_ environment. For cell viability tests using MTT assays (Sigma), cells (1×10^4^) were seeded in quadruplicate in 96-well plates containing medium (200 μl/well) and incubated at 37°C in a CO_2_ incubator. After incubating for 24 h, HCC cancer cell lines in complete medium supplemented with 10% FBS without antibiotics were treated with one of four anticancer drugs at different concentrations: doxorubicin (0.125, 0.25, 0.5, and 1 μg/ml; Sigma), cisplatin (2, 8, 16, and 32 μg/ml; Sigma), 5-fluorouracil (5-FU; 1.25, 2.5, 5, and 10 μg/ml; Sigma) or tamoxifen (2, 4, 6, and 8 μg/ml; Sigma). After a 48 h treatment, cell viability was tested by adding 20 μl of MTT stock solution (5 mg/ml in PBS) to cells and incubating for 4 h under cell culture conditions. The water-insoluble precipitate was dissolved in 100 μl of DMSO and analyzed at 595 nm using an ELISA reader.

### Statistical analysis

Statistical analyses were performed using the SPSS programs (version 18; SPSS Inc., Chicago, IL, USA). Analysis of variance (ANOVA) was used to study the relationship among clinical onset age, alpha-fetoprotein (AFP) levels, tumor grade, tumor size, and gene amplification. Statistically significant differences were defined as those with a *P*-value <0.05. Correlations between copy numbers of the four amplified genes (*CDK13*, *GMNN*, *FAM82B* and *CENPF*) were calculated using Spearman's rank correlation coefficient (*r*).

## Supporting Information

Figure S1 **Differential gene expression analysis of four amplified genes: cancer vs. normal.** The following thresholds were used for analysis: *P*-value (0.01), Fold Change (>2), Gene Rank (top 10%). Cell color was chosen based on the best gene rank percentile for analyses within the cell. Data source: ONCOMINE 4.3 (http://www.oncomine.org/).
(TIF)

Figure S2 **Typical examples of differential gene expression** (**cancer vs. normal**) **presented in Supplementary** **Figure 1** **.** The same thresholds were used for analysis as presented in Supplementary Figure 1. Overexpression of *CENPF* and *GMNN* genes were observed in hepatocellular carcinomas (HCC). In addition, the overexpression of *CDK13* and *FAM82B* genes were also observed in prostate carcinomas. Data source: ONCOMINE 4.3 (http://www.oncomine.org/).
(TIF)

Figure S3 **The increase of gene expression** (**A**) **and cell proliferation assay** (**B**) **in transfected cells.** Stablely transfected cells with candidate genes (*CENPF*, *GMNN*, *CDK13* or *FAM82B*) were screened to measure the increase of gene expression by real-time RT-PCR methods as described in the Materials and Methods. Increase of fold change in gene expression was shown in the figure. *In vitro* cell proliferation of stably transfected cells were examined by MTT assay as described in the Materials and Methods.
(TIF)

Table S1 **SNP-based detection of gene amplification in primary hepatocellular carcinomas.**
(DOCX)

Table S2 **Cell viability assays to test for anticancer drug sensitivity of 11 hepatocellular carcinoma cell lines containing various copy numbers of four amplified genes.**
(DOCX)

Table S3 **Primers used for the real-time PCR.**
(DOCX)
