# Supplementary figures and images for: Frequent Amplification of CENPF, GMNN and CDK13 Genes in Hepatocellular Carcinomas
Source: PLoS One. 2012 Aug 13;7(8):e43223. doi: 10.1371/journal.pone.0043223 (PMC3418236; doi:10.1371/journal.pone.0043223)

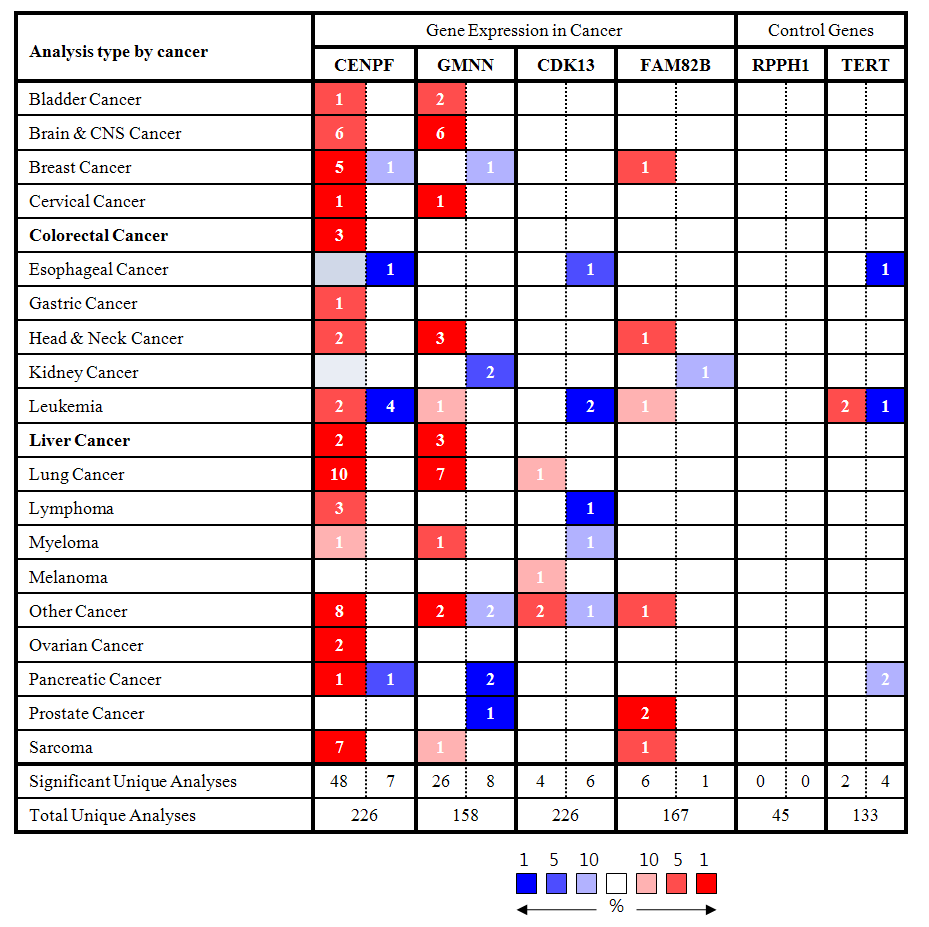

Supplement: Figure S1 — Differential gene expression analysis of four amplified genes: cancer vs. normal. The following thresholds were used for analysis: P-value (0.01), Fold Change (>2), Gene Rank (top 10%). Cell color was chosen based on the best gene rank percentile for analyses within the cell. Data source: ONCOMINE 4.3 (http://www.oncomine.org/). (TIF) [file pone.0043223.s001.tif]

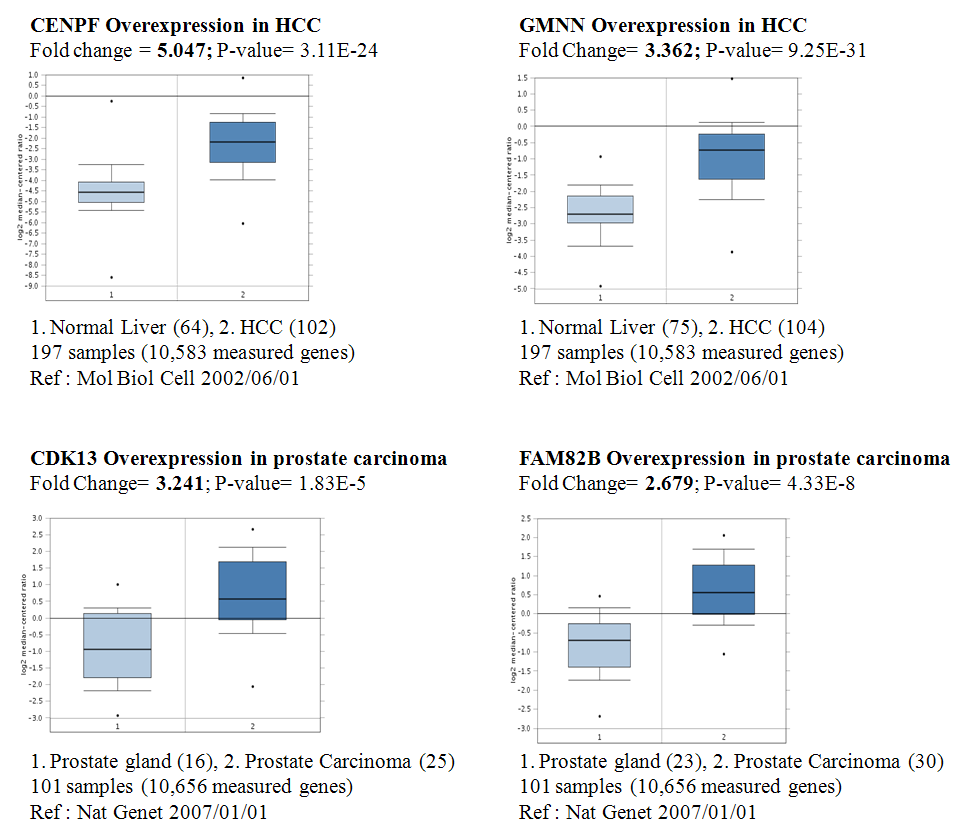

Supplement: Figure S2 — Typical examples of differential gene expression (cancer vs. normal) presented in Supplementary Figure 1 . The same thresholds were used for analysis as presented in Supplementary Figure 1. Overexpression of CENPF and GMNN genes were observed in hepatocellular carcinomas (HCC). In addition, the overexpression of CDK13 and FAM82B genes were also observed in prostate carcinomas. Data source: ONCOMINE 4.3 (http://www.oncomine.org/). (TIF) [file pone.0043223.s002.tif]

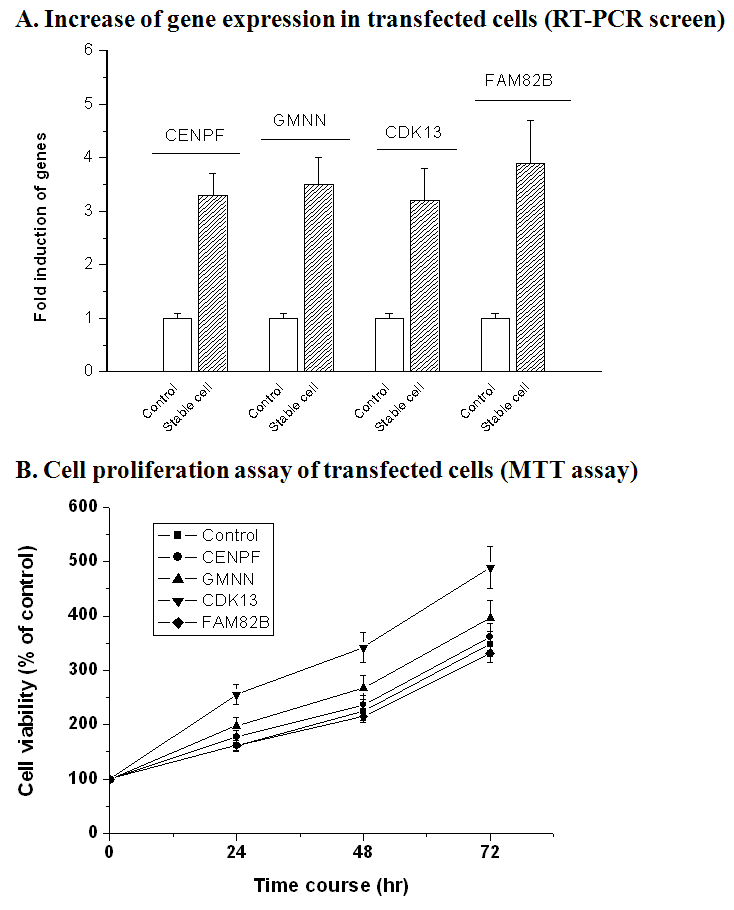

Supplement: Figure S3 — The increase of gene expression (A) and cell proliferation assay (B) in transfected cells. Stablely transfected cells with candidate genes (CENPF, GMNN, CDK13 or FAM82B) were screened to measure the increase of gene expression by real-time RT-PCR methods as described in the Materials and Methods. Increase of fold change in gene expression was shown in the figure. In vitro cell proliferation of stably transfected cells were examined by MTT assay as described in the Materials and Methods. (TIF) [file pone.0043223.s003.tif]
