# Supplementary material for: Frequent Amplification of CENPF, GMNN and CDK13 Genes in Hepatocellular Carcinomas
Source: PLoS One. 2012 Aug 13;7(8):e43223. doi: 10.1371/journal.pone.0043223 (PMC3418236; doi:10.1371/journal.pone.0043223)
Supplement: Table S1 — SNP-based detection of gene amplification in primary hepatocellular carcinomas. (DOCX) [file pone.0043223.s004.docx]

**Table S1.** SNP-based detection of gene amplification in primary hepatocellular carcinomas.

| Locus # | Cytomap | Chr. Position  (hg37.1) |  | rs# (nsSNP) | Gene | No. of samples with gene amplification (n = 12; %) |
| --- | --- | --- | --- | --- | --- | --- |
| 1 | 1q21-41 | 158,584,091 |  | rs952094 | SPTA1 | 4/12 (33.3%) |
|  |  | 169,391,154 |  | rs3820059 | C1orf114 | 4/12 (33.3%) |
|  |  | 171,509,327 |  | rs760644 | PRRC2C | 3/12 (25.0%) |
|  |  | 173,881,122 |  | rs2227606 | SERPINC1 | 4/12 (33.3%) |
|  |  | 183,085,755 |  | rs20563 | LAMC1 | 5/12 (41.7%) |
|  |  | 185,175,837 |  | rs2295950 | C1orf26 | 4/12 (33.3%) |
|  |  | 186,273,994 |  | rs2273779 | PRG4 | 3/12 (25.0%) |
|  |  | 210,577,884 |  | rs2294851 | HHAT | 6/12 (50.0%) |
|  |  | 214,814,733 |  | rs3795519 | CENPF | 5/12 (41.7%) |
| 2 | 6p21-24 | 12,296,217 |  | rs6413478 | EDN1 | 1/12 (8.3%) |
|  |  | 24,780,892 |  | rs1923185 | GMNN | 5/12 (41.7%) |
|  |  | 26,106,237 |  | rs707891 | HIST1H1T | 5/12 (41.7%) |
|  |  | 26,460,721 |  | rs2893856 | BTN2A1 | 6/12 (50.0%) |
|  |  | 27,425,185 |  | rs1883216 | ZNF184 | 5/12 (41.7%) |
|  |  | 28,038,117 |  | rs203881 | ZNF165 | 6/12 (50.0%) |
|  |  | 28,170,297 |  | rs735765 | ZNF193 | 4/12 (33.3%) |
|  |  | 28,240,798 |  | rs1778484 | ZNF187 | 5/12 (41.7%) |
|  |  | 29,934,413 |  | rs3132685 | HCG9 | 5/12 (41.7%) |
|  |  | 30,228,015 |  | rs2844759 | FLJ45422 | 5/12 (41.7%) |
|  |  | 30,810,781 |  | rs2250264 | DDR1 | 3/12 (25.0%) |
|  |  | 31,600,939 |  | rs3130050 | BAT3 | 4/12 (33.3%) |
|  |  | 32,146,526 |  | rs440261 | NOTCH4 | 7/12 (58.3%) |
|  |  | 32,269,251 |  | rs2022544 | C6orf10 | 5/12 (41.7%) |
|  |  | 32,580,992 |  | rs4642516 | HLA-DQA1 | 5/12 (41.7%) |
|  |  | 33,019,005 |  | rs3129270 | COL11A2 | 6/12 (50.0%) |
|  |  | 48,229,606 |  | rs16874954 | PLA2G7 | 2/12 (16.7%) |
| 3 | 7p13 | 40,037,230 |  | rs34775357 | CDK13 | 4/12 (33.3%) |
| 4 | 8q13-23 | 72,984,041 |  | rs16937976 | TRPA1 | 5/12 (41.7%) |
|  |  | 80,567,272 |  | rs4930 | STMN2 | 5/12 (41.7%) |
|  |  | 86,021,932 |  | rs16913589 | LRRCC1 | 3/12 (25.0%) |
|  |  | 87,519,315 |  | rs6980476 | FAM82B | 7/12 (58.3%) |
|  |  | 87,680,282 |  | rs16916632 | CNGB3 | 5/12 (41.7%) |
|  |  | 95,479,680 |  | rs28910279 | RAD54B | 6/12 (50.0%) |
|  |  | 99,044,528 |  | rs2255317 | MATN2 | 7/12 (58.3%) |
|  |  | 110,413,762 |  | rs964307 | PKHD1L1 | 5/12 (41.7%) |
| Total |  |  |  |  | 35 genes |  |

The Human NS-12K SNP chip was used to detect gene amplification in 12 paired hepatocellular tumor tissue samples. For comparison, a total of 90 normal genome samples isolated from the blood of healthy individuals were also genotyped using SNP chips. Gene amplification was detected based on genotype intensities that were more than 1.5-fold higher in tumor samples than in normal samples, with manual checking of each SNP for intensity and the images of genotype cluster plots.
