# Supplementary material for: Frequent Amplification of CENPF, GMNN and CDK13 Genes in Hepatocellular Carcinomas
Source: PLoS One. 2012 Aug 13;7(8):e43223. doi: 10.1371/journal.pone.0043223 (PMC3418236; doi:10.1371/journal.pone.0043223)
Supplement: Table S2 — Cell viability assays to test for anticancer drug sensitivity of 11 hepatocellular carcinoma cell lines containing various copy numbers of four amplified genes. (DOCX) [file pone.0043223.s005.docx]

**Table S2.** Cell viability assays to test for anticancer drug sensitivity of 11 hepatocellular carcinoma cell lines containing various copy numbers of four amplified genes.

| Drug\Gene | CENPF | | | | GMNN | | | | CDK13 | | | | FAM82B | | | |
| --- | --- | --- | --- | --- | --- | --- | --- | --- | --- | --- | --- | --- | --- | --- | --- | --- |
|  | Loss  (n= 0) | Normal (n=4) | Gain (n=7) | *P* | Loss (n=1) | Normal (n=8) | Gain (n=2) | *P* | Loss (n=1) | Normal (n=5) | Gain (n=5) | *P* | Loss (n=1) | Normal (n=5) | Gain (n=5) | *P* |
| Doxorubicin (1 μg/ml) |  | 68±8.29 | 68±6.33 | 0.635 | 67 | 67±6.35 | 75±7.78 | 0.115 | **63** | **64±3.78** | **73±6.95** | **0.046** | 63 | 69±6.88 | 68±7.57 | 0.916 |
| Cisplatin (32 μg/ml) |  | 63±4.72 | 64±4.81 | 1.000 | 66 | 63±4.91 | 66±4.95 | 0.513 | 63 | 64±3.05 | 64±6.57 | 1.000 | 63 | 64±6.16 | 63±3.78 | 0.753 |
| 5-FU (10 μg/ml) |  | **58±3.50** | **64±5.39** | **0.044** | 63 | 61±6.09 | 66 | 0.112 | 56 | 63±6.99 | 62±4.42 | 0.752 | 56 | 59±3.51 | 65±5.94 | 0.114 |
| Tamoxifen (8 μg/ml) |  | 70±10.23 | 70±3.04 | 0.340 | 69 | 71±7.00 | 68±0.71 | 0.359 | **63** | **74±6.78** | **68±2.86** | **0.045** | 63 | 71±8.11 | 70±3.44 | 0.751 |

MTT assay values, presented as mean ± standard deviation, are expressed as a percentage of control fluorescence. The numbers of copies of the four amplified genes were determined by TaqMan-CNV assay. Statistical significances were evaluated using Mann-Whitney tests. Significant *P*-values are shown in bold.
