# Supplementary material for: Frequent Amplification of CENPF, GMNN and CDK13 Genes in Hepatocellular Carcinomas
Source: PLoS One. 2012 Aug 13;7(8):e43223. doi: 10.1371/journal.pone.0043223 (PMC3418236; doi:10.1371/journal.pone.0043223)
Supplement: Table S3 — Primers used for the real-time PCR. (DOCX) [file pone.0043223.s006.docx]

**Table S3.** Primers used for the real-time PCR.

| Gene Name | Forward Primer | Reverse Primer |
| --- | --- | --- |
| CENPF | 5'-TTGTAAAGAAAGGGTTTGC-3' | 5'- CCAGCTGTTGGTTTGGAGG -3' |
| GMNN | 5'- CGGGCGAGCGGAGTTAGCAG -3' | 5'- TGGCTGCAGCACCTCGCAAA -3' |
| CDK13 | 5'- AAGCAGAAGCCCGTATTCATC -3' | 5'- GCTGCCAGGCTACTCTTCAG -3' |
| FAM82B | 5'- CTTCTGCCTTTCCGACGTG -3' | 5'- GTTTCCCATTACCTCGAAGCC -3' |
| S18 | 5'- TTTGCGAGTACTCAACACCAACA -3' | 5'- CCTCTTGGTGAGGTCAATGTCTG -3' |
